# Anticancer potential of decursin, decursinol angelate, and decursinol from *Angelica gigas* Nakai: A comprehensive review and future therapeutic prospects

**DOI:** 10.1002/fsn3.4376

**Published:** 2024-07-31

**Authors:** Simona Sestito, Roberta Ibba, Federico Riu, Sara Carpi, Antonio Carta, Clementina Manera, Solomon Habtemariam, Balakyz Yeskaliyeva, Zainab M. Almarhoon, Javad Sharifi‐Rad, Simona Rapposelli

**Affiliations:** ^1^ Department of Chemical, Physical, Mathematical and Natural Sciences University of Sassari Sassari Italy; ^2^ Department of Medicine, Surgery and Pharmacy University of Sassari Sassari Italy; ^3^ Department of Chemistry−BMC Uppsala University Uppsala Sweden; ^4^ NEST, Istituto Nanoscienze‐CNR and Scuola Normale Superiore Pisa Italy; ^5^ Department of Health Sciences University ‘Magna Græcia’ of Catanzaro Catanzaro Italy; ^6^ Department of Pharmacy University of Pisa Pisa Italy; ^7^ Pharmacognosy Research & Herbal Analysis Services UK University of Greenwich Kent UK; ^8^ Faculty of Chemistry and Chemical Technology Al‐Farabi Kazakh National University Almaty Kazakhstan; ^9^ Department of Chemistry, College of Science King Saud University Riyadh Saudi Arabia; ^10^ Department of Biomedical Sciences College of Medicine, Korea University Seoul Republic of Korea; ^11^ Centro de Estudios Tecnológicos y Universitarios del Golfo Veracruz Mexico

**Keywords:** anticancer, decursin, decursinol, natural product, traditional medicine

## Abstract

Many naturally derived compounds are currently used in oncotherapy. Besides official medicine, complementary and alternative medicine practices, including old herbal remedies, are widely used and accepted as additional tools in cancer treatment. *Angelica gigas* Nakai (AGN), a medicinal herb in Asia, has roots historically used in medicine. This review focuses on key bioactive compounds from AGN roots – decursin, decursinol angelate (DA), and decursinol (DOH). Exploring their source, biosynthesis, and therapeutic mechanisms, the review highlights their role in cancer treatment. Biotechnological strategies for enhanced production and semisynthetic derivatives with anticancer properties are discussed. The study emphasizes the promising pharmacological potential of decursin, DA, and DOH in various therapeutic applications, particularly cancer treatment. The review also underscores innovative approaches to increase production and explores semisynthetic derivatives as a promising avenue for future natural product‐based drug discovery. This concise overview provides valuable insights into the potential of AGN‐derived compounds in the field of natural product‐based therapeutics.

## Graphical abstract

Decursin and its pyranocoumarin analogs decursinol angelate (DA) and decursinol (DOH) are the most important bioactive compounds isolated from *Angelica gigas* Nakai (AGN) roots, a medicinal herb historically used in Asiatic traditional medicine. Decursin and its analogs interact with different targets involved in cancer development and progression or in related dysfunctions, such as angiogenesis or inflammation. With the aim of improving selectivity and potency of decursin, several semisynthetic derivatives were designed and synthesized often starting with the DOH scaffold.

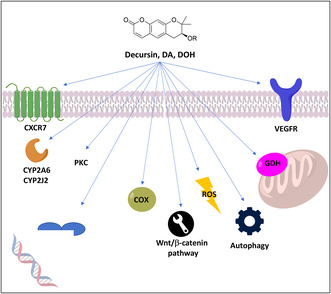

## 1 INTRODUCTION

For millennia, nature has served as the main reservoir of bioactive molecules. In the last few centuries, large number of microbes, plants, and other living organisms have been extensively investigated in the attempt to detect pharmacological activity often as part of ethnomedicinal studies. Big progresses were also made in isolating novel natural products (NPs), and in manipulating them to obtain new nature‐inspired pharmacological agents. Drug discovery from natural sources has always been challenging, mostly due to several factors including the hard‐large‐scale isolation, the difficulty in revealing mechanistic profile, and the dependence on natural source which is often difficult to access. Therefore, many pharmaceutical companies worldwide have reduced their investments in this field in favor of other drug discovery approaches such as structure‐based design. More recently, also, the availability of innovative technologies has revitalized the interest in NPs and synthetic derivatives, allowing to expand the knowledge in their complex pharmacology and increasing both isolation and drug discovery efficiency. From a therapeutic point of view, the pleiotropic profile of NPs offers a unique opportunity to treat multifactorial diseases still unresolved, such as age‐associated neurodegenerative diseases and cancer. In the case of cancer, it was estimated that 1 in 4 anticancer drugs approved from 1981 to 2019 traced their origin to NPs (Newman & Cragg, 2020; Sharifi‐Rad et al., 2019). Famous examples are the phytoalkaloids: paclitaxel, etoposide, irinotecan, and vincristine; and the antibiotics: actinomycin D, mitomycin C, and bleomycin. Despite severe side effects, most of them are still used in cancer therapy today due to the lack of more efficacious treatment options (Huang et al., 2021).

Besides modern medicine, complementary and alternative medicine (CAMs) practices have always been of help to fight cancer worldwide. Among them, old herbal remedies, used in Asia for thousands of years, are widely used and accepted as additional tools in cancer treatment. Low costs and lack of serious toxicity make these preparations a suitable choice to treat cancer and other chronic disorders.

**FIGURE 1:**
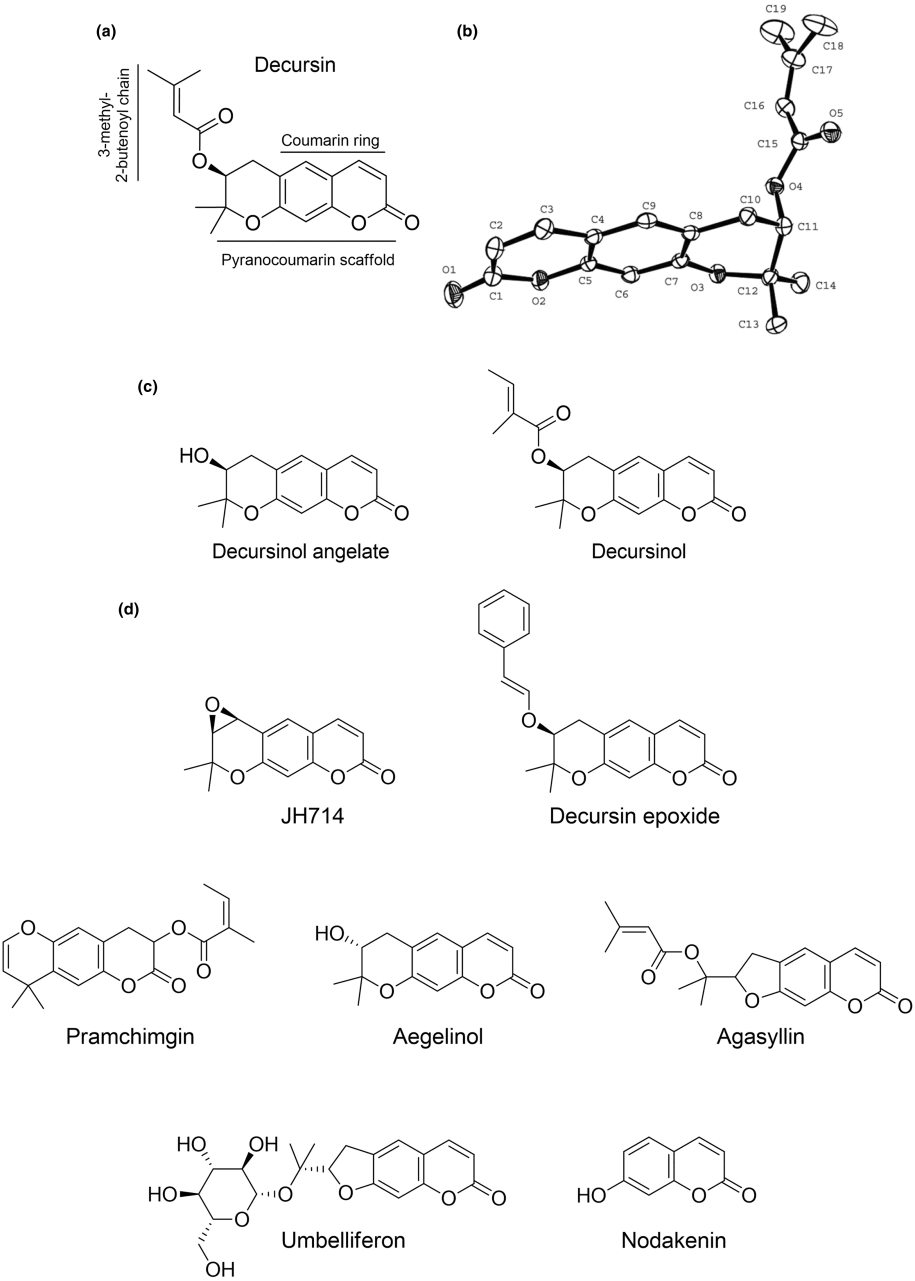
The (a) 2D and (b) 3D structures of decursin. (c) Chemical structures of decursinol angelate and decursinol. (d) Some of the minor pyranocoumarin‐based compounds found in AGN roots.

*Angelica gigas* Nakai (AGN) belongs to the Umbelliferae family. Widely growing in many Asian countries, such as Korea, Japan, and China, the roots of this plant have been traditionally used to treat hormonal imbalance and anemia (Kang et al., 2022), and for liver detoxification (Kwon et al., 2021). Decursin is one of the most important bioactive compounds isolated from AGN roots, together with linear pyranocoumarin analogs, including decursinol angelate (DA), decursinol (DOH), epoxide decursin, oxime decursin, diketone decursin, and other simple and furanocoumarin compounds, such as umbelliferone and nodakenin (see Figure 1; Önder, 2020; Shehzad et al., 2018). This review focused our attention on decursin and its principal metabolites DA and DOH, summarizing their main natural source, the biosynthetic pathway, the molecular and cellular mechanisms underlying the chemopreventive and other anticancer activities, the current medical applications, as well as some biotechnological studies oriented to improve their production.

## 2 REVIEW METHODOLOGY

Most of the data were sourced using the search keywords “decursin,” “decursinol,” “decursin/DA/DOH + anticancer,” and “decursin/DA/DOH + safety/toxicity” in Google Scholar and PubMed with no limitation of time. Clinical trials were searched via clinicaltrials.gov. Only studies focusing on decursin, DA, and DOH targets involved in cancer development and progression or in related pathological markers such as angiogenesis or inflammation were included. Semisynthetic derivatives were collected by using Reaxys tool. Only derivatives for cancer treatment were described. For completeness, semisynthetic derivatives for nononcological therapeutic applications were reported in Table 1.

## 3 *Angelica gigas* NAKAI (AGN) PLANT

Although pyranocoumarin (see Figure 1) compounds are produced by numerous members of the *Angelica* species, decursin and DA are mainly obtained from the roots of *Angelica gigas* Nakai (AGN). AGN is a plant of the family of Apiaceae (Umbelliferae). This family includes annual, biennial, or perennial herbs and grows in Asia, Europe, and North America. More specifically, AGN is a monocarpic biennial or short‐lived perennial plant that inhabits forests, banks of streams, and grasslands, and grows in different countries across East Asia, including Korea, Japan, and northeastern China (Park et al., 2020; Shehzad et al., 2018). The plant grows to a height of 1–2 m with a ribbed and purplish stem. AGN shows purple double umbrella‐shaped flowers that bloom between August and September and has many small rootlets on the thick roots (Park et al., 2020).

The Forum for the Harmonization of Herbal Medicines (FHH), that is, the government network for traditional medicine in the Western Pacific Region, published in July 2018 an interesting report on AGN where it is exhaustively described the external and internal morphology of the radix and the portion of pharmacological interest. According to the *Monograph of the Korean Pharmacopeia* (in Korean Pharmacopeia Tenth 2016 Edition Monographs_Part 2. Tenth Edition ed., 1259), the plant is described as follows: AGN root is conical or narrow long conical in shape, usually branched, 15–25 cm in length, and 2–5 cm in diameter. The external surface fades from pale yellowish brown to blackish brown with irregular longitudinal wrinkles and spot‐shaped remains of fibrous roots. The crown is broad, usually with remains of stems and leaves. The texture is hard, but fragile. The fractured surface has a pale brown or yellowish brown cortex, relatively sparse with numerous clefts, and the xylem is yellowish white or white. Under a microscope, the transverse section reveals a cork consisting of five to six layers of cells aligned transversely, parenchyma from the primary cortex to xylem aligned systemically in oval or rectangular shapes. The phloem has cambium and schizogenous intercellular space, secretory canals with yellowish brown ingredients, and sparsely scattered bast fiber bundles. Scalariform or spiral vessels are observed. In parenchyma cells, there are many starch grains. This root has a thin, distinctive odor, and a little bitter and sugary taste (Reddy et al., 2017).

Finally, a note is also necessary around natural source of DA and DOH. Sometimes AGN, also called Korean Dang Gui, is confused with *Angelica sinensis*, named Dang Gui in China. As a result of similar names, appearances, and shapes, it is not uncommon for them to be confused with each other (Su‐Jin et al., 2022) For these reasons, novel classification strategies, using HPLC and other methods, are under development to ensure an appropriate and correct identification of the natural source without mistake (Jeong et al., 2015; Su‐Jin et al., 2022).

## 4 STRUCTURE AND GENERAL CHARACTERIZATION OF DECURSIN AND DOH

(+)‐Decursin (D, C_19_H_20_O_5_, MW = 328 Da) is one of the most abundant linear dihydropyranocoumarin compound in nature. It was first isolated from the root of *Angelica decursiva* Fr. et Sav. (Hata & Sano, 1969) and later from *A. gigas* Nakai roots (Konoshima et al., 1968). Decursin is also among the various coumarins in *A. gigantis*, a plant used as an intestine moistener, for abdominal pain and traumatic injuries (Hwang, 1998). The 3D structure of decursin was studied by Shin et al., using X‐ray crystallography (Shin et al., 2008). It showed a geometric conformation represented by an L‐shape with a hinge in C11. As shown in Figure 1a, the carbonyl oxygen and the planar coumarin scaffold have a 10 Å distance between each other. The condensed dihydropyran ring is slightly distorted in a half‐chair conformation, with a 3‐methyl‐2‐butenoyl chain in axial position, which was unusual for a bulky group, and almost rectangularly oriented. The axial orientation is explained by the steric hindrance generated by the C13 and C14 methyl groups as geminal substituents.

Another pyranocoumarin, DA (C_19_H_20_O_5_, M_w_ = 328 Da), identified as decursin isomer, was abundantly found in AGN root as well (Ahn et al., 2008). A consistent amount of decursin and DA were also identified in the ethanol extract of *A. tenuissima* (Korean “Gubon”; Islam et al., 2009) and *A. glauca* Edgew roots (Saeed & Sabir, 2008). In addition, decursin and DA were isolated from the roots of *Saposhnikovia divaricata*, a traditional Chinese herb (Zhao et al., 2010). For the sake of completeness, decursin and DA were reported in other traditional polyherbal medicines, such as Ka‐mi‐kae‐kyuk‐tang (KMKKT), Bangpungtongsung‐san, Sanghwa‐tang (SHT), LMK02‐Jangwonhwan, Sipjundaebo‐tang, and Ojeok‐san (OJS), employed for the treatment of various disorders such as obesity, inflammation, fever, amnesia, neuralgia, rheumatism, hyperlipidemia, and other diseases (Zhang, Li, et al., 2012).

Two precursors of decursin, DOH and its enantiomer aegelinol (Figure 1), were also isolated from the aerial parts of AGN (Reddy et al., 2008). DOH is a direct analog of decursin (C_14_H_14_O_4_) with MW = 246 Da, where the (CH_3_)_2_‐C=CH‐COO‐ side chain is simplified with a hydroxyl (‐OH) group. It can be identified with a much lower abundance compared to decursin and DA (Ahn et al., 1997, 2008). It was also found to be far less active than decursin (Yim et al., 2005). Interestingly, a protocol to produce DOH by mild basic hydrolysis of decursin and DA was introduced (Li et al., 2012; Zhang, Shaik, et al., 2012). The structures of decursin, DA, and DOH have been confirmed by UV, IR, MS, ^1^H‐NMR, ^13^C‐NMR, and optical rotation by different groups (Ahn et al., 1996, 1997; Hata & Sano, 1969; Xia et al., 2009). Figure 1 reports 2D and 3D structures of decursin, chemical structures of DA and DOH, and some minor pyranocoumarin‐based compounds found in AGN roots.

### 4.1 *Angelica gigas* and its coumarin constituents

*Angelica gigas* Nakai (AGN) is a Korean plant belonging to the *Angelica* L. genus within the Umbelliferae family which contains more than 60 species (Kim, Kim, & Kang, 2009; Xia et al., 2009). It was found in Korean moist soil, but it is recognized as a traditional medicine mainly in Korea, China, and Japan (Jiangtao & Chongren, 2004; Sarker & Nahar, 2004). The plant itself has been originally employed as a tonic and for the treatment of several diseases, such as anemia (Chi & Kim, 1970), but also as a sedative or as an anodyne (Oh et al., 2017). Some additional effects were also attributed, including antibacterial (Lee, Shin, Kim, et al., 2003) acetylcholinesterase inhibitory activity (Bihel et al., 2003), myocardial relaxant activity, protein kinase C activity (Ahn et al., 1996), and anticancer activity against sarcoma cancer cells (Lee, Lee, Jung, et al., 2003). AGN was deeply investigated for its chemical composition (Kim, Kim, & Kang, 2009; Song et al., 2011). Decursin was originally identified as the main ingredient found in AGN roots, stems, and leaves (Jiangtao & Chongren, 2004). Besides DA, other decursin‐related compounds are the benzylethyl ether JH714, and the epoxide derivative, chemically ((6*S*,7*R*)‐6,7‐epoxy‐8,8‐dimethyl‐7,8‐dihydro‐6*H*‐pyrano‐[3,2‐g]chromen‐2‐one; Lee et al., 2006; Mahat et al., 2013). Anticancer (Lee, Lee, Jung, et al., 2003), antibacterial (Lee, Shin, Kim, et al., 2003), neuroprotective (Kang & Kim, 2007), and antiplatelet (Lee, Lee, Jin, & Yun‐Choi, 2003) effects were mainly attributed to these pyranocoumarin‐based derivatives. Other minor pyranocoumarin components are DOH and prantschimgin (Jiangtao & Chongren, 2004). Noteworthy, decursin, DA, and DOH present a chiral carbon that links the hydroxyl group in DOH or the side chain in decursin and DA. Notably, aegelinol and agasyllin, the enantiomers of (+)‐DOH and (+)‐DA, respectively, exist naturally in AGN (Jung & Huneck, 1991). The cited pyranocoumarins are reported in Figure 1.

Interestingly, the diameter size of AGN root can influence the content and amount of its main components, decursin and DA. Their total amount increases as the root diameter is thinner. The so‐called thin root part of AGN has a higher amount of decursin and DA, which leads to a higher antioxidant activity compared to other root parts (Lee, Lee, Jin, et al., 2019). In 2019, a study was conducted on the weight characteristics and the compounds' content of AGN, comparing plants from four cultivation areas in Pyeongchang (South Korea). The dry weight was the highest in the Tapdong‐ri group (13.37 ± 0.13 g) and the lowest in the SangjinBu‐ri group (11.90 ± 0.57 g). The content of bioactive compounds was the highest in the Tapdong‐ri group (116.13 ± 9.53 mg/g) and the lowest in the SangjinBu‐ri group (99.77 ± 23.23 mg/g; Jeong et al., 2019). Hence, the content of these compounds could depend on where the plant grows in Korea or other geographical locations.

### 4.2 Conventional extraction of pyranocoumarin‐based compounds from AGN

Conventional methods, such as steam distillation and hydrodistillation, percolation, maceration, or extraction in Soxhlet apparatus with organic solvents, were employed for the isolation of compounds before their GC–MS characterization (Kim et al., 2006; Kim & Lee, 2002; Richter et al., 2005). Using these methods, several compounds have been extracted and identified from AGN, particularly decursin (Konoshima et al., 1968) and DA (Ryu et al., 1990). The first approach was the extraction with different solvents. In 2002, in Korea, Sanghyun Lee et al. isolated decursin and DA from 5 kg of air‐dried powdered roots of AGN, together with other substances such as bergapten, nodakenetin, uracil, and nodakenin. The material was extracted three times with MeOH under reflux. The extracts were combined and concentrated, resulting in 1125 g of crude product which was suspended in water and fractioned with equal volumes of Et_2_O and *n*‐BuOH. The fractions were evaporated in vacuo, resulting in 518 g from the Et_2_O fraction and 445 g from the *n*‐BuOH one. Decursin, DOH, bergapten, and nodakenetin were obtained via silica gel column chromatography from the Et_2_O fraction (Yim et al., 2005), while the *n*‐BuOH one gave other compounds including nodakenin and uracil. The isolated compounds from both fractions were then characterized by NMR 400 MHz and ESI‐MS (Lee et al., 2002).

As for the content weight of the extracted components, a study on the methanol extracts of AGN reported the following abundances for the main pyranocoumarins (mg/g): decursin (18.7–44.8), DA (11.1–36.8), DOH (0.083–0.431), demethylsuberosin (0.412–2.31), marmesin (0.059–0.592), and nodakenin (4.53–13.1; Ahn et al., 2008). The major pyranocoumarins which can be extracted from methanol (i.e., decursin, DA and DOH) may constitute 3.0%–8.2% of the air‐dried weight of AGN root (Kang et al., 2005). A high‐performance liquid chromatography–mass spectroscopy (HPLC‐MS) characterization of decursin and DA was performed, giving *m/z* = 329 [M + H]^+^ and *m/z* = 351 [M + Na]^+^ as MS results, on >95% pure material from HPLC (60% EtOH, −20°C for 12 h; Kim, Jung, et al., 2009).

Moreover, pyranocoumarins were also characterized by HPLC/DAD (high‐performance liquid chromatography with diode‐array detection), considering that DAD method for quantitative analysis of marker compounds and pattern recognition analysis can provide a good prospect for the comprehensive quality control of AGN and other members from *Angelica* genus. A mixture of water and acetonitrile was used as the mobile phase with the addition of 0.1% formic acid for better peak symmetry and shape. All target compounds and internal standards were completely detected in 60 min at 325 nm with maximum absorption (Jeong et al., 2015).

**FIGURE 2:**
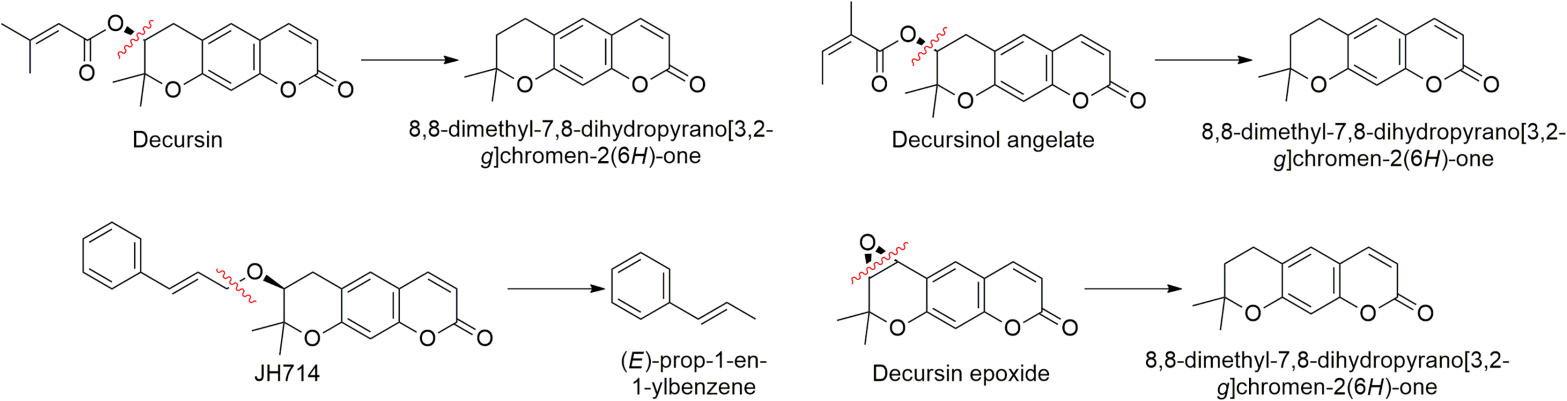
MS fragmentations pattern of the main pyranocoumarins found in AGN (Mahat et al., 2013).

Some other fragments which can be identified through mass spectrometry for some of the most important pyranocoumarins are reported in Figure 2 (Mahat et al., 2013).

Due to the hydrophobicity, decursin can be extracted by ethanol or supercritical CO_2_ fluid, but not by water (Zhang, Li, et al., 2012). The yield of decursin could be increased in the microwave‐assisted extraction of AGN roots through the optimization of parameters such as ethanol concentration, microwave power, and extraction time (Kim & Lee, 2002; Zhang, Li, et al., 2012). More effective ways to extract bioactive compounds from AGN were investigated. The most common were hydrothermal, ethanol, and supercritical CO_2_ extraction methods. This latter strategy was found to be the most effective, with a content of 38.65% of decursin and DA in the extracts (Park et al., 2021). A brief summary of the extraction and analysis methods used to identify pyranocoumarin‐based compounds in AGN is reported below.

Other extraction methods used to identify pyranocoumarin‐based compounds in AGN are: (a) pressurized liquid extraction, better known as accelerated solvent extraction (ASE), which allows faster extractions with small volumes of solvents (Giergielewicz‐Możajska et al., 2001; King, 2000); (b) ionic liquids, as an alternative to volatile organic solvents (Duffy et al., 2012); (c) subcritical water extraction (SWE), an environmentally friendly approach which allows higher extraction yield, greater purity of the extract, and lower disposal costs (Ravber et al., 2015). These methods have been used to extract AGN derivatives with variable yields (Cho et al., 2007; Kiyonga et al., 2019; Ko et al., 2020).

## 5 BIOSYNTHESIS OF DECURSIN, DA, AND DOH

**FIGURE 3:**
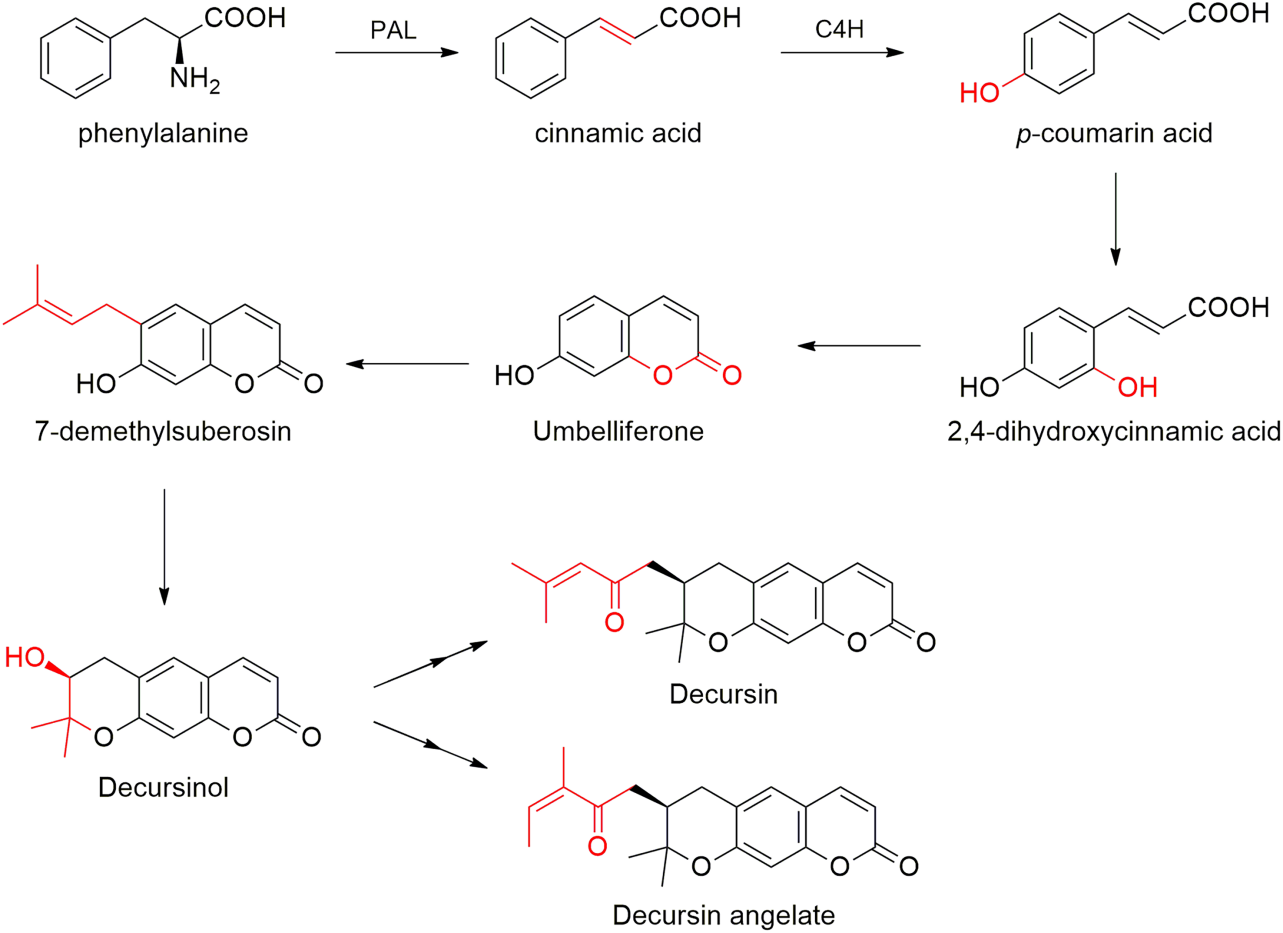
Proposed biosynthetic pathway of decursin, DA, and DOH in AGN. PAL: phenylalanine ammonia‐lyase; C4H: cinnamate 4‐hydroxylase (modified from Park, Park, Xu, & Park, 2010). The portion of the molecule subjected to biochemical modification is evidenced in red.

The biogenesis of decursin, DA, and DOH has been extensively investigated. As with the source of many physiologically relevant plant metabolites (Dixon & Paiva, 1995), the phenylpropanoid pathway is central to the biosynthesis of decursin and related chemicals. This biosynthesis pathway is also the route of synthesis for aromatic amino acids such as phenylalanine (Phe) which yields cinnamic acid by the action of phenylalanine ammonia‐lyase (PAL). Another important enzyme is cinnamate 4‐hydroxylase (C4H) which adds a hydroxyl group at the C‐4 or *para*‐position of the phenyl ring of cinnamic acid to yield *p*‐hydroxy‐coumaric acid. Further hydroxylation of the aromatic ring generates the 2,4‐dihydroxylcinnamic acid. The latter hydroxylation is crucial for the formation of the coumarin scaffold, leading to umbelliferone. Finally, the functionalization with a 2‐methylbut‐2‐ene chain (7‐demethylsuberosin) and the subsequent cyclization to linear pyranocoumarin yields DOH. DOH can be further functionalized by giving the two isomeric derivatives decursin and DA. Figure 3 depicts the entire biosynthetic pathway leading to the formation of decursin DA and DOH in AGN roots. This proposed biosynthesis pathway was further supported by experiments using precursors labeled with stable radioisotope in the hairy root culture of AGN (Ji et al., 2008).

## 6 BIOTECHNOLOGICAL STRATEGIES TO INCREASE DECURSIN AND DOH PRODUCTION

Considering the wide therapeutic potential of decursin, DA, and DOH, some researchers focused on the development of innovative biotechnological strategies aimed to enhance their metabolic production. For instance, Park, Park, Lee, et al. (2010) and Xu et al. (2008) reported two protocols for the establishment of transgenic AGN root cultures using different strains of *Agrobacterium rhizogenes*, the most studied bacterium able to transfer genes to higher plants. Both methods led to a significant increase in decursin content in hairy roots.

As previously discussed, phenylalanine ammonia‐lyase (PAL) and cinnamate 4‐hydroxylase (C4H) are crucial enzymes for the biogenesis of decursin/DA/DOH (Rhee et al., 2010). In the attempt to prompt the production of DA, the coumarin biosynthetic pathway was engineered and transgenic hairy roots overexpressing PAL and C4H genes were developed. It is interesting to note that the C4H but not the PAL‐transgenic hairy root culture could produce more DA than the control the groups (Park et al., 2012).

Interestingly, Fontamillas and colleagues demonstrated that laying hens fed with the dried AGN powder (root, stem, and leaf) produced eggs with decursin and DA in white and yolk. Moreover, the plasma cholesterol levels of hens fed with AGN powder were significantly decreased. The authors concluded that AGN can be introduced as feed in the poultry industry to improve the quality of eggs and health of animals (Fontamillas et al., 2019).

## 7 DECURSIN SEMISYNTHETIC DERIVATIVES

With the aim of improving selectivity and potency of decursin, several semisynthetic derivatives were designed and synthesized. The new molecules were often designed by starting with the DOH scaffold that possesses an easily functionalizable free hydroxy group (Figure 1). The new compounds were demonstrated to interfere with diverse cellular pathways leading to diverse pharmacological effects (Table 1). Here, we limited the discussion to those possessing a documented anticancer activity.

**FIGURE 4:**
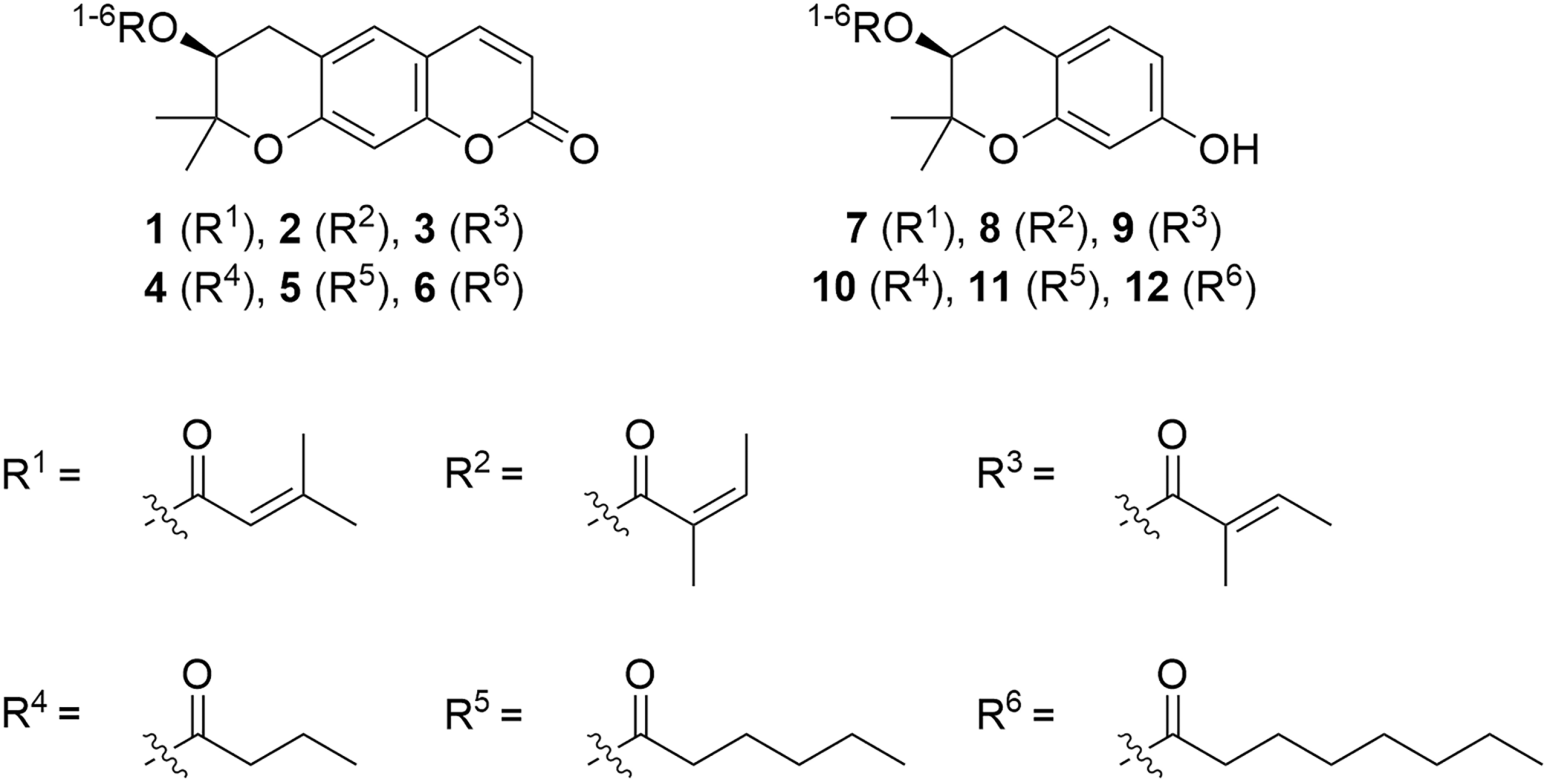
Decursin (**1**) and derivatives **2**–**12** synthesized by Kim et al. (2005).

Decursin, DA, and DOH were proven to promote protein kinase C (PKC) activation, thereby showing remarkable anticancer activity. In 2005, DOH was chosen by Kim and colleagues as the lead compound for the design and synthesis of more effective anticancer agents endowed with PCK modulation activity (Kim et al., 2005). Kim et al. reported the synthesis of decursin and 11 derivatives depicted in Figure 4. The first class of compounds kept the dihydropyranocoumarin main scaffold, while the second class possessed a dimethylchromanol scaffold obtained by skeleton simplification of the previous one. The side substituents were in turn selected among the isomeric unsaturated moieties seleciolate (R^1^), angelate (R^2^), *cis*‐angelate (R^3^) all bearing a double bond, or saturated aliphatic chain with three (R^4^), five (R^5^), or seven (R^6^) carbon atoms (Figure 4). Decursin (**1**) and compounds **2**–**6** were proven to act as tumor‐suppressing agents against leukemia cells, while only Decursin (**1**) and derivatives **2** and **3** turned out to be cytotoxic against TUR, a resistant myeloid‐leukemia cell line. The sole compounds **2** and **3**, together with parental compound **1**, acted as PKC βII‐dependent antiproliferative agents, while derivatives **4**–**6** showed a PKC βII‐independent mechanism of action, implying the side chain is the structural moiety that dictates activity toward PKC βII. In general, structure–activity relationship (SAR) analysis based on the cytotoxic and mechanism‐of‐action (MoA) investigation revealed that the coumarin main scaffold is mandatory for the antiproliferative activity and the side chain is crucial for the PKC recruitment and the cytotoxic molecular mechanism (Kim et al., 2005).

**TABLE 1:** Decursinol semisynthetic derivatives with nononcological activity.

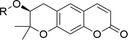			
Original label	R	Biological activity	Ref
KC1	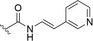	Suppressive activity on HMGB1‐mediated septic responses via HMGB1 release reduction	Lee, Yuseok, Lee, et al. (2019)
KC2	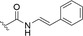		
KC3	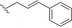		
JH‐4	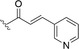	Suppressive activity on HMGB1‐mediated septic responses via HMGB1 release reduction	Lee, Yuseok, Yang, et al. (2019)
		Hutchinson–Gilford progeria syndrome phenotype alleviation via inhibition of progenin–lamin A/C binding	Lee, Jung, et al. (2016)
JH‐1	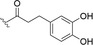	Hutchinson‐Gilford progeria syndrome phenotype alleviation via inhibition of progenin–lamin A/C binding	
JH‐13	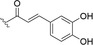	Hutchinson‐Gilford progeria syndrome phenotype alleviation via inhibition of progenin–lamin A/C binding	
Cmpd 6		Alleviation of the pathogenesis of lung tissue damage in the asthmatic mouse model via leukocytosis and eosinophilia in lung tissue inhibition	Yang et al. (2009)
ALA‐DA	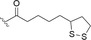	Neuroprotective effects against ischemic damage via decrease of glial activation	Lee et al. (2012)
ASA‐DA	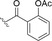	Neuroprotective effects against transient focal and global cerebral ischemic damage via attenuation of glial activation and maintenance of antioxidants	Yan et al. (2013)
Cmpd 3	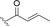	Antirheumatic activity via Bruton's tyrosine kinase (BTK) inhibition	Cho et al. (2020)
Cmpd 4	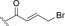		
Cmpd 7	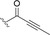		
Cmpd 8	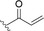		
Cmpd 9	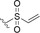		
Cmpd 10	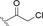		
LKY‐047	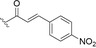	Selective inhibitor of 2J2 isoform of cytochrome P450	Phuc et al. (2017)
Cmpd 4	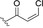	Antifungal activities against various plant disease pathogens via selective inhibition of mycelial growth and spore germination	Shin et al. (2022)
Cmpd 5	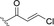		
Cmpd 11	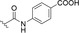	Antidiabetic activity via blood glucose reduction	Joo et al. (2017)
Cmpd 15	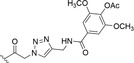	Anti‐Alzheimer's disease activity via AChE inhibition	Anand et al. (2012)
Cmpd 11	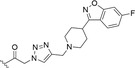	Anti‐Alzheimer's disease activity via both AChE and BuChE inhibition	
Cmpd 12	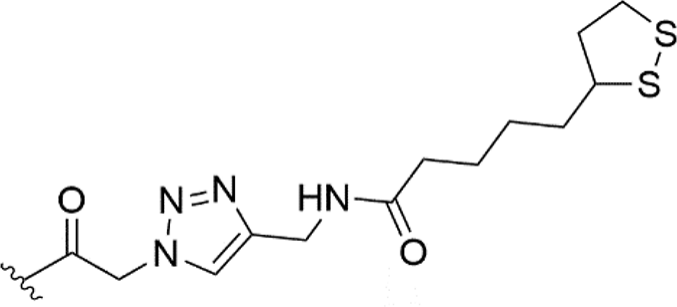	Anti‐Alzheimer's disease activity via BuChE inhibition	
Cmpd 13	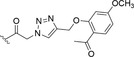		

**FIGURE 5:**
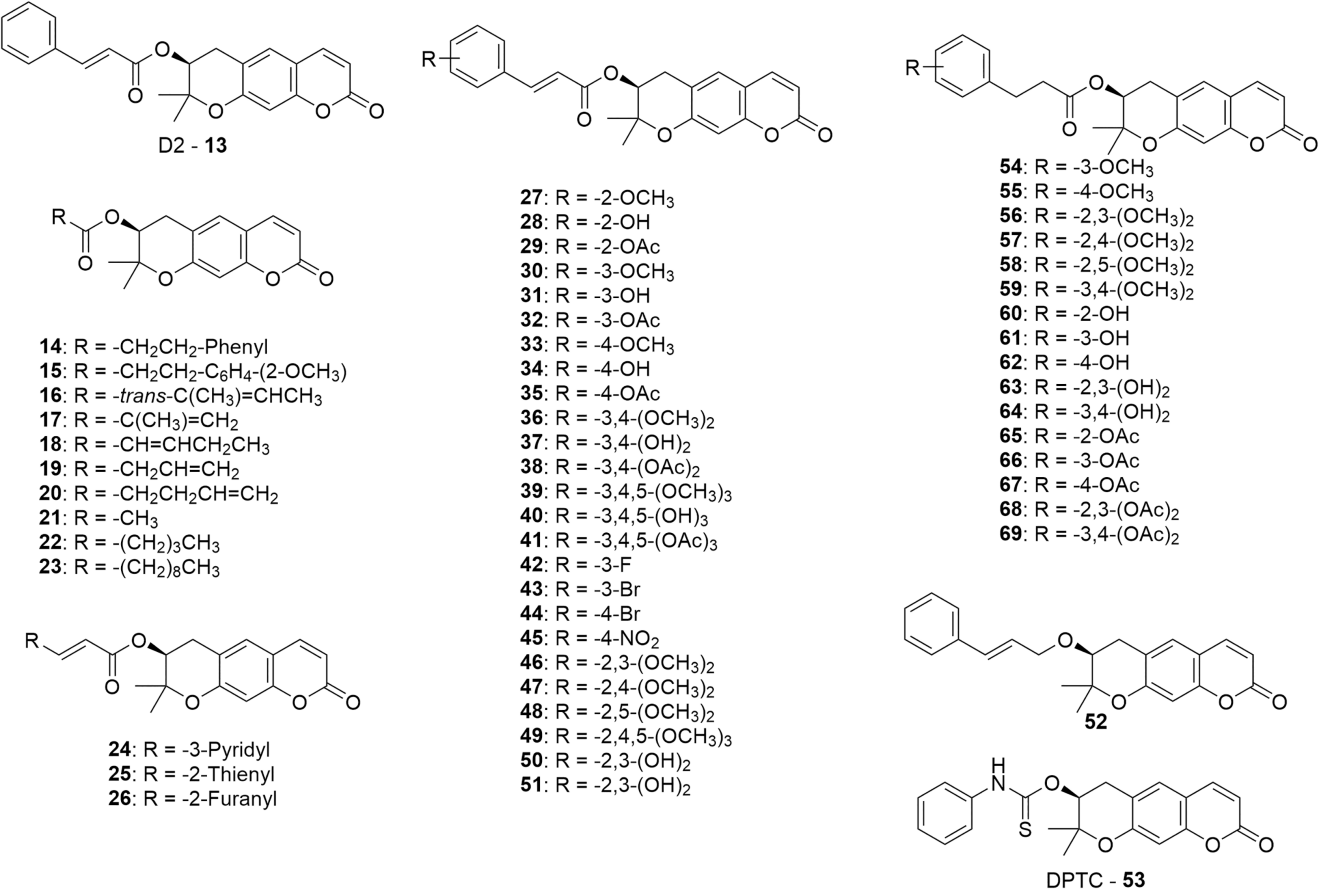
Chemical structure of dihydropyranocoumarin D2 (**13**), semisynthetic derivatives synthesized (reported by Lee et al. (2010) and Lee, Kim, et al. (2016)), and decursinol phenylthiocarbamate (DPTC, **53**; Zhang, Shaik, et al. (2012)).

Lv et al. (2007) showed that AGN extract acts as hypopigmenting agent in B16 melanoma cells by reducing isobutylmethylxanthine‐induced melanogenesis. A considerable dose‐dependent suppression of melanin production was detected at the concentration ranging from 5 to 30 μg/mL of *Angelica* Radix extracts, without any cytotoxic effect. Moreover, the downregulation of tyrosinase expression, the major melanosomal enzyme involved in melanogenesis, was noticed. These results are particularly relevant to dermatological disorders which are characterized by an abnormal deposition of melanin. Hence, decreasing melanin production by AG extracts could exert a therapeutic effect (Lv et al., 2007). In 2010, Kim and collaborators tested both DOH and its synthetic derivative D2 (**13**, Figure 5) in Mel‐Ab melanocyte cell line to evaluate the potential hypopigmentary activity. D2, tested at 1–100 μM concentrations, strongly inhibited melanogenesis without any cytotoxicity. To investigate the MoA, the tyrosinase activity was assessed: interestingly, no direct inhibitory activity was detected in the cell‐free system, but tyrosinase levels were downregulated and their activity decreased as a function of the concentration (Kim, Park, et al., 2010). Also, extracellular signal‐regulated kinase (ERK), Akt activation, and microphthalmia‐associated transcription factor (MIFT) have a great influence on the synthesis of melanin and were therefore measured in Mel‐Ab cells after D2 administration. While Akt was not affected, ERK turned out to be drastically upregulated, resulting in the reduction of melanin production. MITF levels were decreased, comparable to tyrosinase protein levels after treatment with D2. These results suggested that the hypopigmentary activity of compound **13** at 50 μM is related to the downregulation of both MITF and tyrosinase (Kim, Park, et al., 2010). Lee et al. (2010) reported the synthesis and inhibitory activity of a series of 30 DOH‐based compounds, obtained by *O*‐alkylation or esterification with various side chains (Figure 5). MTT assay showed no relevant cytotoxic effect at the active concentrations for most of the 30 compounds. Then, inhibition of melanin synthesis was evaluated in B16 melanoma cells, using decursin, DA, and DOH as control. While decursin and DA (both DOH esters) showed melanin synthesis inhibition, the parent DOH demonstrated poor inhibitory activity. These preliminary results suggested *O*‐functionalization as a tool for potency improvement. Compounds **16** and **18**, bearing an alkenyl substitution on DOH hydroxyl group, displayed an increased potency coupled with a moderate cytotoxicity. Alkenoyl groups were then replaced with alkanoyl moieties, gaining, among others, the two most potent compounds **22** and **23**, which showed a melanin inhibition rate of 76.5% and 79.3% at 100 μM, respectively, still jointly with moderate cytotoxicity (10.4% and 28.2%, respectively). The following round of side chain modifications produced a series of acrylic acid derivatives (compounds **13**, **24**–**41**) which secured several nontoxic but with highly potent melanin inhibitors (derivatives **13, 25**, and **27**). Despite displaying appreciably inhibitory activity ranging from 65.9% to 80.7% at 100 μM, compounds **28**, **29**, **31**, **32**, **34**, and **35** bearing a free hydroxy group (‐OH) or an acetylated one (‐OAc) in position 2, 3, or 4 of the phenyl rings showed high undesired cytotoxicity. Ultimately, compound **52** possessing the ether linker turned out to be a weak inhibitor suggesting that the presence of ester moiety is crucial for the efficacious reduction of melanin synthesis in B16 cells (Lee et al., 2010).

The androgen receptor (AR) is a validated target for the prevention and treatment of prostate cancer (PCa), and androgen deprivation via pharmaceutical treatment induces remission of the primary form of PCa (Scher & Sawyers, 2005). Jiang and colleagues proved that decursin and DA are potent in vitro AR signaling inhibitors (Jiang et al., 2006) by a different molecular mechanism if compared with the control compound bicalutamide (Lü et al., 2007). At the same time, when decursin and DA are administered in vivo, both compounds are rapidly hydrolyzed to DOH, which showed remarkably less potent inhibitory activity (Lee, Kim, et al., 2016). On these bases, Zhang et al. theorized that DOH derivatives with nonhydrolyzable and therefore more stable moieties may show higher potency also in vitro. As a consequence, they designed and synthesized the DOH phenylthiocarbamate (DPTC, **53**, Figure 5) and evaluated the in vivo stability and the inhibitory activity against AR signaling both in vitro and in vivo (Zhang, Shaik, et al., 2012).

The in vivo stability of DPTC **53** was evaluated by HPLC after i.p. injection in mice: the plasma peak of the highest concentration was revealed 30 min after injection and it decreased in the following 60 minutes, suggesting conjugation and rapid excretion. Nonrevealable plasma concentrations of DOH were also measured at all timepoints. When decursin and DA were administered, the plasma peak rapidly disappeared to make rise the DOH peak, confirming the unstable nature of the ester compounds. This confirmed the hypothesis that thiocarbamate moiety is more metabolically stable when compared to the ester one (Zhang, Shaik, et al., 2012). The DPTC MoA was deeply investigated in LNCaP cells, and it was uncovered that DPTC induces a reduction of AR‐dependent transcription and AR nuclear translocation; downregulation of AR protein was also detected along with mRNA concentration reduction. These findings suggested that the metabolic stabilization of the DOH derivatives side chain would represent a great strategy to develop new series of potent, stable, and selective anti‐AR agents derived from DOH, useful for the prevention and treatment of PCa (Zhang, Shaik, et al., 2012).

**FIGURE 6:**
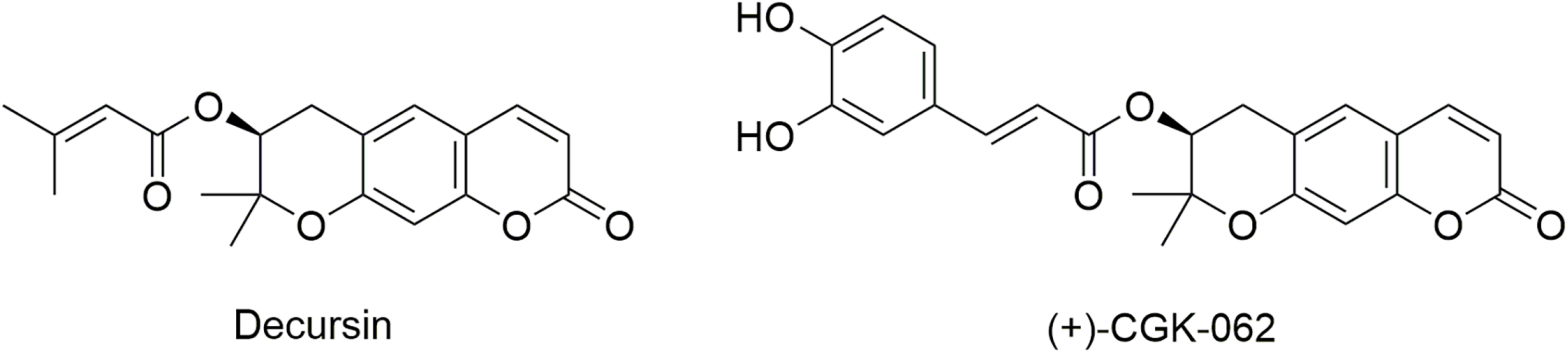
Chemical structure of decursin and (+)‐CGK062.

Over the years, different coumarin‐based compounds such as decursin and (+)‐CGK062 (**37**; Figure 6) were found to be efficacious in the inhibition of the Wnt/β‐catenin pathway, thus inducing tumor suppression such as colon and prostate cancer. For instance, this pathway was proved to be activated in PC3 prostate cancer cells by abnormal upregulation of cytoplasmic β‐catenin. Therefore, Lee and colleagues designed and synthesized two series of DOH‐based compounds bearing a cinnamoyl or phenyl propionyl side chains, using DOH as starting material and compound (+)‐CGK062 as the lead compound. They reported the synthesis of some already known DOH derivatives along with similar new ones, as shown in Figure 5 (Lee, Kim, et al., 2016).

A cell‐based screening assay for potential Wnt/β‐catenin pathway inhibition clearly showed the cinnamoyl‐substituted compounds as more potent inhibitors when compared to the phenyl propionyl ones. These results suggest that the double bond in the side chain is crucial for the interaction and therefore inhibition of the Wnt/β‐catenin pathway. Also, the substitution position on the phenyl ring showed to be crucial and *ortho‐* or *meta‐*functionalized compounds turned out to be the most potent. Derivative **32**, bearing a 3‐acetoxy cinnamoyl moiety in the side chain, was selected by the authors as the best compound since it showed inhibition activity comparable to lead compound (+)‐CGK062. Interestingly, (+)‐CGK062 contains two hydroxyl groups on the lateral phenyl ring which appeared to be responsible for the conjugation with glucuronic and/or sulfuric acids, and its rapid in vivo excretion. Molecular mechanism investigation performed on HEK293 cell lines proved that compound **32** inhibited the Wnt/β‐catenin pathway by reducing the β‐catenin level. Furthermore, compound **32** was proved to reduce the expression of cyclin D1 and c‐myc, known β‐catenin target genes, suppressing PC3 cell growth in a dose‐dependent manor. Therefore, hit compound **32** represents a promising derivative for its appreciable inhibitory activity against prostate cancer and its advantageous manipulation of phenolic groups in acetyl ones.

**FIGURE 7:**
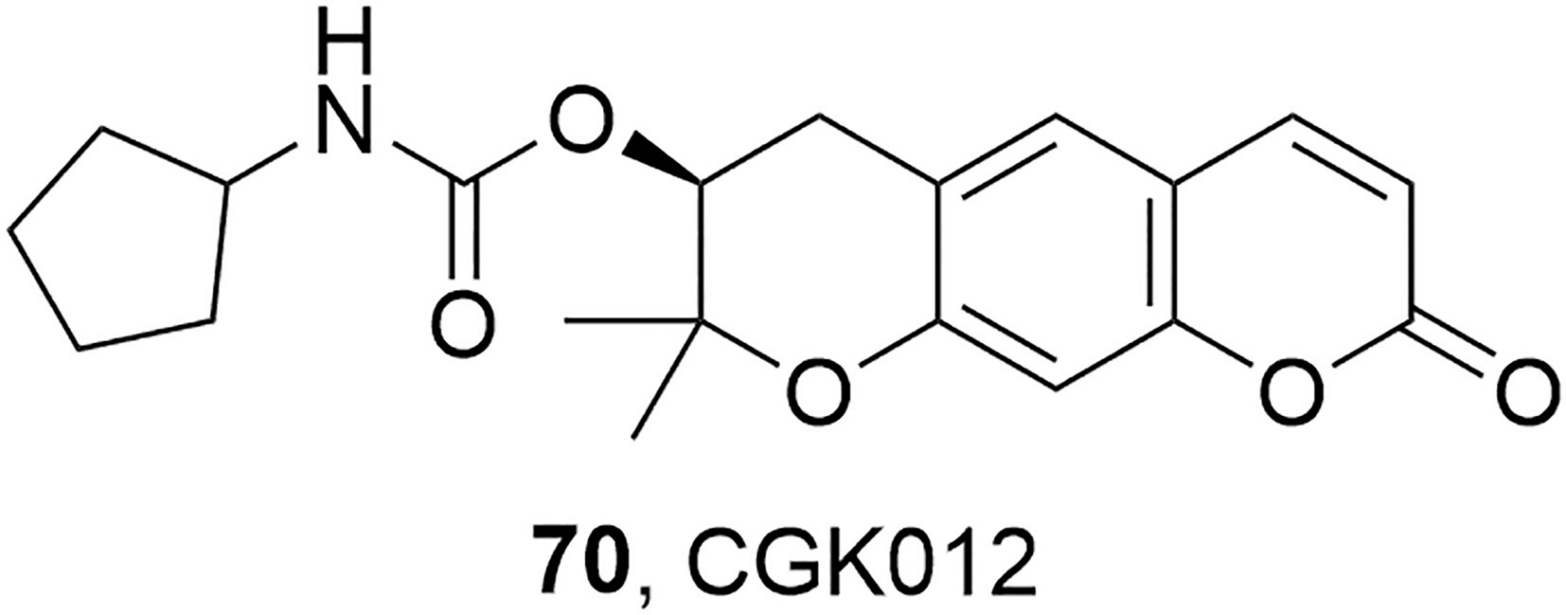
Chemical structure of compound CGK012 (**70**; Jun Choi et al., 2017).

Notably, the Wnt/ β‐catenin pathway inhibition was also studied by Choi et al. for its antiproliferative effect also in multiple myeloma cells. In these cells, the Wnt pathway is activated, and the concentration of intracellular β‐catenin is constitutively high (Jun Choi et al., 2017). In their work, the authors synthesized the carbamate **70**, named CGK012 (Figure 7), obtained by coupling DOH with cyclopentyl isocyanate. CGK012 was assessed for its inhibitory activity against Wnt/β‐catenin pathway in genetically engineered HEK293‐FL cells presenting an induced overexpression of β‐catenin. As a result, compound **70** was able to decrease the catenin response transcription and to lower the intracellular levels of β‐catenin, coupled with any detectable cytotoxicity. These results suggested that derivative **70** was capable of suppressing the Wnt/β‐catenin pathway in vitro by inducing proteasomal degradation of β‐catenin in the cell, representing a good starting point to develop new optimized DOH‐based antineoplastic agents for the treatment of multiple myeloma (Jun Choi et al., 2017).

## 8 MECHANISMS OF ANTITUMOR ACTION OF DECURSIN, DA, AND DOH

**FIGURE 8:**
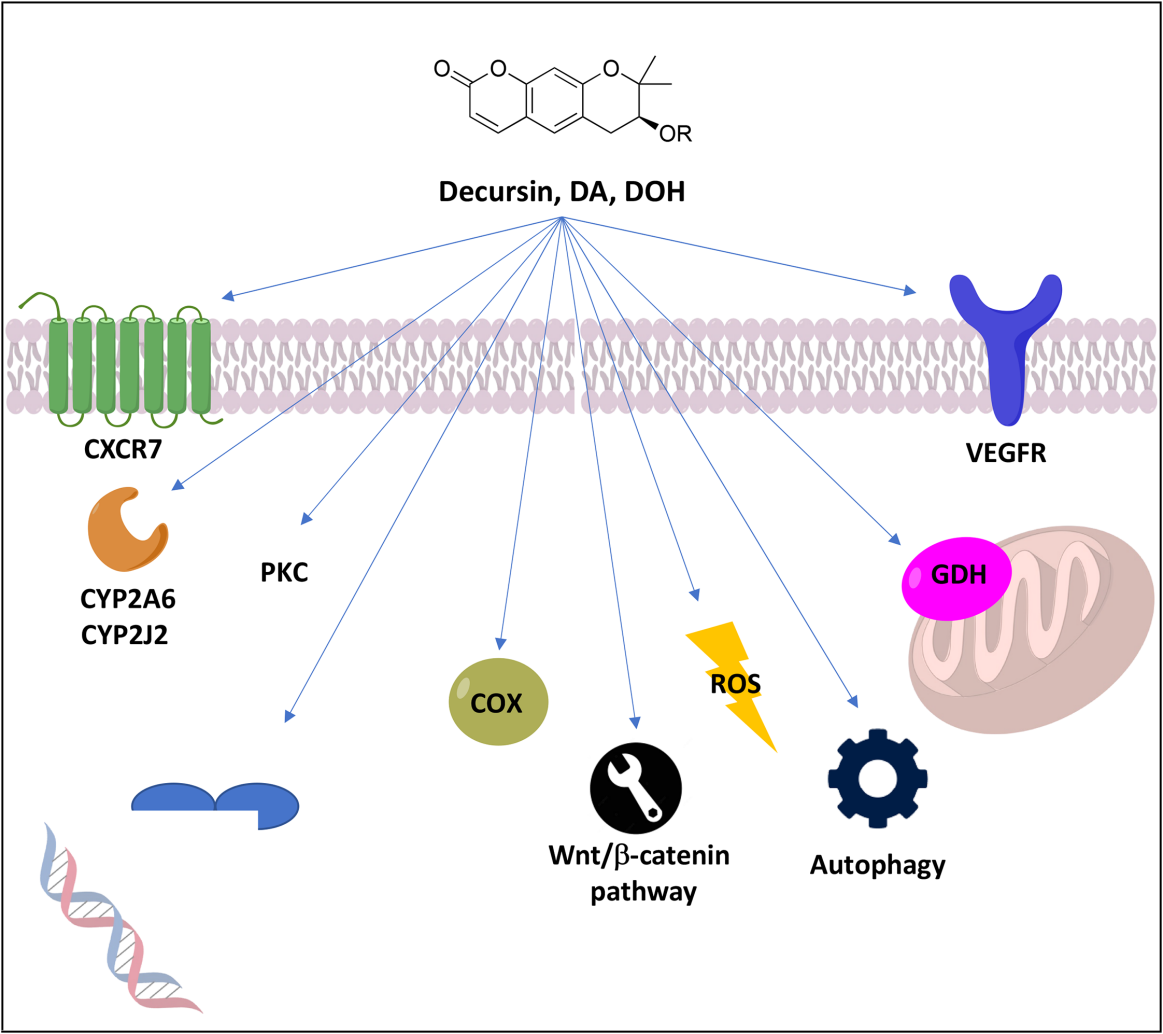
Decursin, DA, and DOH main targets involved in cancer development and progression or in related dysfunctions. Through several mechanisms resume below: protein kinase C (PKC) and ROS modulation; hypoxia‐inducible factor 1α degradation; chemokine receptor CXCR7 downregulation; glutamate dehydrogenase (GDH) inhibition; reduction of the expression of cyclooxygenase‐2 enzyme (COX2); vascular endothelial growth factor receptor (VEGFR) inhibition; downregulation of CYP2A6 expression; and/or CYP2J2 modulation.

As already highlighted, decursin, DA, and DOH were present in some traditional herbs used for different therapeutic applications, including many chronic disorders. Therefore, these NPs were widely investigated to reveal their molecular mechanism, and the relevant activities have been reported. The first report of in vitro cytotoxic activity in cancer cell lines for decursin and DA was published in 1996 (Ahn et al., 1996, 1997), while in vivo studies on sarcoma‐180 ascitic tumor mice followed in 2003 (Lee, Lee, Jung, et al., 2003). Since then, many studies reporting in vitro (Kim, Lee, et al., 2010; Son et al., 2011) or in vivo (Lü et al., 2022) anticancer activity have been published. Here, we decided to focus our attention on the main decursin, DA, and DOH targets involved in cancer development and progression or in related dysfunctions, such as angiogenesis or inflammation (Figure 8).

### 8.1 Protein kinase C

PKC family are serine/threonine kinases involved in the signal transduction for cellular proliferation and differentiation through phosphorylation of downstream effectors. PKC family includes 15 isozymes classified into three groups: conventional, novel, or atypical based on their second messenger requirements. Prolonged cellular exposure to PKC activators may trigger downregulation or degradation of PKC isoforms. Ahn et al. were the first group reporting the cytotoxic effect (with ED_50_ values lower than 20 μg/mL) of decursin against human cancer cell lines including leukemia, breast cancer, gastric carcinoma, hepatocarcinoma, and melanoma cell lines. Interestingly, the authors linked this biological activity to PKC and reactive oxygen species (ROS) modulation (Ahn et al., 1996). Some years later, decursin and other synthetic derivatives were investigated in K562 leukemia cells at 50 μM concentration, demonstrating an antiproliferative effect triggered by PKC activation. Moreover, decursin was proved to induce the downregulation of PKCa and bII, previously activated by the tumor‐promoting PKC activator PDBu (Kim et al., 2005). More recently, a study was carried out on breast cancer metastasis obtained by the administration of the selective PKC activator 12‐*O*‐tetradecanoylphorbol‐13‐acetate (TPA). The results evidenced the blockade of MCF‐7 invasion triggered by decursin. Molecular mechanism investigation enlightened the involvement of PKCα together with MAPK and NF‐κB pathways, and their common downstream target matrix metalloproteinases 9 (MMP9; Kim et al., 2014).

### 8.2 Hypoxia‐inducible factors (HIFs)

HIFs are transcription factors sensible to oxygen deficiency. HIFs are heterodimeric complexes of two subunits: the oxygen‐dependently regulated subunit α and the constitutively expressed subunit β. Under normoxic conditions, both subunits are hydroxylated by prolyl hydroxylases (PHDs), ubiquitinated by von Hippel Lindau E3 ubiquitin ligase (VHL), and then degraded by proteasomes. Otherwise, hypoxic conditions inactivate PHDs, then promoting HIFs transcriptional activities. Additionally, the level of α subunits can be modulated by several oncogenic factors such as the PI3K/AKT/mTOR pathway, the phosphatase and tensin homolog enzyme (PTEN), and the signal transducer and activator of transcription 1 (STAT1). Activated HIFs transcriptionally control a broad range of genes, resulting in apoptosis suppression, cancer aggressiveness, metastasis, drug resistance, and anticancer immunity limitation. Several phytochemical classes, including alkaloids, organosulfurs, terpenes, and polyphenols demonstrated to influence HIF behavior (Yun et al., 2021). Decursin promotes HIF‐1α degradation in A549 human non–small‐cell lung carcinoma cells and in human colorectal HCT116 cancer cells under hypoxic conditions. They showed transcriptional suppression of target genes, such as C‐X‐C motif chemokine receptor 4 (CXCR4) and vascular endothelial growth factor (VEGF); decursin also promoted apoptosis and decreased cell invasion. In Lewis lung carcinoma (LLC) allograft murine model, decursin (10 mg/kg, 7 days of treatment) improved T‐cell activation, thereby enhancing the anticancer immune response in tumor microenvironment (TME; Ge et al., 2020).

### 8.3 Glutamate dehydrogenase (GDH, GLUD1)

GDH is a mitochondrial hexameric NADPH‐dependent enzyme whose function is to catalyze the reversible oxidative deamination of glutamate to α‐ketoglutarate and ammonia. This conversion supports cancer survival since it furnishes intermediates to the Krebs cycle, contributing to the energetic balance. A very recent work by Chang et al. (2022) described a combined in silico/in vitro investigation of the binding of decursin and DA to GDH. In this article, the authors provided statistical data showing that African American and Caucasian populations were more susceptible to early mortality due to high GDH expression in colorectal cancer patients. Then, through docking and molecular dynamics studies, they showed that decursin and DA bind GDH in a similar conformation to the standard reference inhibitor epigallocatechin gallate (EGCG), but with higher binding affinity. Finally, enzymatic GDH inhibition assays confirmed the predicted activity showing a higher potency of the two pyrocoumarins (IC_50_ values of 1.035 μM for decursin and 1.432 μM for DA) when compared to EGCG (1.94 μM; Chang et al., 2022).

### 8.4 Cyclooxygenase (COX)

COX enzymes are mostly known as the main regulators of inflammation; however, they are responsible for multifactorial effects in various tissues. While COX‐1 has a constitutive expression, COX‐2 is inducible, and its upregulation promotes cancer and inflammatory diseases; therefore, re‐equilibration of COX‐2 level is considered as a potential approach for the prevention/therapeutic treatments of inflammation and related diseases (Shehzad et al., 2018). For instance, COX‐2 promotes cell survival and inhibits apoptosis in chronic myelogenous leukemia (CML), a disorder characterized by abnormal differentiation and proliferation of myeloid cells in bone marrow. Decursin was able to reduce the COX‐2 expression in CML in a concentration‐dependent manner, thus reducing CML development. Additionally, decursin inhibits other proinflammatory mediators largely produced in the TME, such as chemokines and cytokines, contributing to the anticancer and anti‐inflammatory effects (Ahn et al., 2010).

### 8.5 Vascular endothelial growth factor receptor (VEGFR)

VEGFRs are tyrosine‐kinase receptors activated with high affinity by endogenous VEGF. Once activated, VEGFRs trigger several intracellular signal transductions to induce angiogenesis, lymphangiogenesis, vasculogenesis, and cancer development. Blocking VEGFR signal might alter the TME, which sustains cancer growth. Moreover, VEGF seems to be crucial also for tumor metastasis and VEGFR inhibitors are frequently applied in metastatic cancer (Liu et al., 2011). Decursin and DA inhibit VEGF‐induced angiogenic processes in human umbilical vein endothelial cells (HUVEC), blocking proliferation, migration, and tube formation. Investigations in chicken eggs confirmed the in vitro evidence, since decursin and DA suppressed neovessel formation in chorioallantoic membrane. Subcutaneous administration of 4 mg/kg/day in the Lewis lung cancer mice model significantly reduced tumor growth and microvessel density compared to the control. MoA investigation in endothelial cells indicated the inhibition of VEGF‐dependent VEGFR2 autophosphorylation, with consequential inhibition of the downstream phosphorylated effectors p42/44 ERK and JNK–MAPK. MAPK activation by VEGFR2 phosphorylation further triggers matrix metalloproteinase (MMP) activation and potentiation of endothelial cell migration; coherently, inhibition of VEGF‐induced MMP‐2 activation was also observed (Jung et al., 2009). A later study reports decursin‐induced vasculogenesis inhibition by showing its ability to attenuate endothelial progenitor cells (EPC) differentiation and function in neovessels in the same mice model (Jung et al., 2012).

### 8.6 Autophagy

Autophagy flux is a crucial mechanism for the degradation of intracellular components which participates in the regulation of normal cell life. The role of autophagy in a cancer setting is still ambiguous: initially, it was indicated as tumor suppressor via degradation of cancer cells, but mounting evidence has been suggested to promote tumor survival and progression in conditions of stress. Coherently, decursin has been demonstrated to inhibit autophagy and counteract gastric cancer in SNU216 and NCI‐N87 cell lines, slowing tumor growth and inducing cell cycle arrest. This effect was demonstrated by the detection of a reduced expression of lysosomal protein cathepsin C (CTSC), which, in turn, led to the accumulation of the autophagic markers LC3 and SQSTM1. Simultaneous inhibition of E3F3, a cell cycle regulatory transcription factor, contributes to the global anticancer effect. Interestingly, the antitumor effect was proved in vivo in spheroid and patient‐derived gastric organoid models, confirming the altered expression of CTSC and autophagy‐related proteins (Kim et al., 2021).

### 8.7 CXC chemokine receptor 7 (CXCR7)

CXC chemokine receptor 7 (CXCR7) is a seven‐transmembrane G‐protein‐coupled receptor activated by the chemokines CXCL11/I‐TAC and CXCL12/SDF‐1CXCR7. Chemokines are a family of small, secreted cytokines which regulate a variety of cell functions. When the C‐X‐C motif chemokine ligand 12 (CXCL12) binds to C‐X‐C chemokine receptor type 4 (CXCR4) and C‐X‐C chemokine receptor type 7 (CXCR7), their downstream signaling pathways mediate broad effects on chemotaxis, cell proliferation, migration, and gene expression. For instance, CXCR7 is suggested not only to regulate cell migration and survival and neurogenesis (Chen et al., 2015), but it also intervenes in heart and kidney development (Haege et al., 2012). On the other hand, accumulating data indicated the CXCL12/CXCR4/CXCR7 axis as coresponsible also in tumor development, survival, angiogenesis, metastasis, and chemoresistance. Thus, CXCR4/CXCR7 modulators have been developed and used for preclinical and clinical cancer treatments (Shi et al., 2020). CXCR7 engagement in decursin anticancer activity has been revealed in two different tumor sceneries. In gastric cancer, CXCR7 increases cell proliferation, growth, migration, and invasion via the upregulation of STAT3/c‐Myc signaling. Decursin inhibits cancer growth and induces apoptosis by decreasing the expression of CXCR7 and the downstream STAT3 pathway. Moreover, apoptotic induction due to the reduction of antiapoptotic factors such as Bcl‐2 was observed both in gastric cancer cell and in tumor xenograft (Kim et al., 2019). Similar effects were registered in head and neck squamous cell carcinoma (HNSCC): decursin attenuates protumorigenic effect of CXCR7 inducing its downregulation (Joo et al., 2022). All these results confirm the anticancer activity of decursin and its capability to counteract CXCR7‐dependent cancer progression.

### 8.8 Wnt/β‐catenin

The Wnt/β‐catenin pathway plays a key role in cell proliferation and differentiation, but it is also associated with embryonic development. Deregulation of this pathway leads to increased β‐catenin‐dependent transcription, contributing to tumor development in different districts like the prostate one. With regard to the interaction between decursin and Wnt/β‐catenin signaling, divergent studies evidenced contrasting activities. A cell‐based screening on prostate cancer cells evidenced the ability of decursin to inhibit PC3 growth via degradation of β‐catenin, thus antagonizing the β‐catenin response transcription (CRT) and suppressing the expression of the downstream targets cyclin D1 and c‐Myc. Interestingly, none of the effects induced by decursin were observed with DOH administration. These findings suggest an anticancer activity in prostate cancer for decursin, which completely disappears for DOH (Song et al., 2007). In contrast, decursin and DA proved to suppress adipogenesis through the activation of the β‐catenin signaling pathway (Park et al., 2019). Although deregulated Wnt/β‐catenin signaling is one of the main genetic alterations in human hepatocellular carcinoma (HCC), this MoA has not been reported for the effect observed in the article of Li, Wang, et al. (2018) who examined the effects of decursin on the growth of HepG2 cells.

### 8.9 CYP2A6 and CYP2J2 isoforms

CYP2A6 and CYP2J2 belong to the P450 family, notoriously involved in phase I‐dependent metabolism through NADPH‐dependent redox reactions. Their abnormal function could result in the altered metabolism of toxic species. For instance, CYP2A6 plays a crucial role in the metabolism of carcinogens from tobacco to genotoxic intermediates; DA downregulates CYP2A6 in human liver microsomes as well as reduces the effect of nicotine in the smoker's body, but also contrasts nicotine intake in smokers (Yoo et al., 2007). On the other side, CYP2J2 mediates the production of endogenous metabolites' epoxyeicosatrienoic acids (EETs) from arachidonic acid; both CYP2J2 and EET are involved in cancer settings. In HepG2 cells, decursin showed a cytotoxic effect together with a reduction of CYP2J2 activity without modification of its expression, suggesting a beneficial effect in hepatic cancer (Lee et al., 2014).

### 8.10 Oxidative stress

ROS are known to contribute to cancer onset and progression, but they also play a prominent role in inflammatory and neurodegenerative settings. As with many other NPs, pyrocoumarins from AGN exert an antioxidant activity helpful in counteracting these processes (Prasad et al., 2017). In a recent article focusing on AGN extract, decursin has been shown to exert an antioxidant activity and affect the 5′‐adenosine monophosphate‐activated protein kinase (AMPK) pathway (Song et al., 2022). Although neuroprotective activity for decursin was demonstrated in an in vitro PC12 model of neuroinflammation (Li, Yang, et al., 2018), antioxidant activity of decursin DA and DOH in cancer settings has not yet been explored and represent a potential field for further investigation.

## 9 METABOLISM AND OTHER PHARMOKINETICS ASPECTS

Decursin, DA, and DOH were discovered to possess a different metabolic route, whose knowledge is quite crucial. A detailed report on AGN extract and its signature phytochemicals decursin, DA, and DOH was recently published (Lü et al., 2022). The latter review, which analyzes in‐depth pharmacokinetics (PK) and metabolism of three compounds (decursin, DA, and DOH), underlined the hypothesis of rapid and extensive hydrolyzation of decursin and DA to DOH due to hepatic first‐pass metabolism by CYP isoforms 2C19 and 3A4.

In 2011, liquid–liquid extraction and HPLC‐UV analyses were used by Li et al. (2012) to set up a practical method to quantify decursin, DA, and DOH in mouse plasma as well as tumor tissues from nude mouse xenografts. The study revealed that the majority of both decursin and DA are hydrolyzed to DOH in their mouse models. In rat models, Park et al. (2012) only detected DOH in plasma, indicating that decursin underwent substantial first‐pass hepatic degradation and had a very low bioavailability.

Zhang et al. (2015) tested decursin and DA for stability in rodents' plasma and whole blood, they found a slight conversion of decursin/DA into DOH; on the contrary, when decursin and DA were orally administered, a fast in vivo conversion into the less active DOH was always detected. Then, aiming at the identification of metabolism site and drove by Park et al. (2012) results, which suggest a liver metabolism, they tested in vitro decursin and DA stability in S9 fractions from mouse and rat liver and intestine. S9 fractions contain several phases I and II enzymes. They measured 95% of decursin/DA conversion into DOH after 15 minutes incubation with liver S9 fractions, while only trace amounts of DOH were detected when decursin and DA were incubated for 30 minutes with intestine S9 fractions. These results provided more evidence that the liver, rather than the intestine, is the key organ where decursin and DA are hydrolyzed.

Notably, PK properties of the three compounds present slight differences translating from in vitro to animals and humans. Even though humans seem to metabolize DA slightly slower than rodents, also data from human administration indicate an extensive hydrolysis of decursin and DA to DOH. On this basis, it could be assumed that in vivo results regarding anticancer efficacy, safety, tissue distribution, and pharmacodynamic biomarkers could be taken as reference for human studies (Zhang et al., 2015).

However, DOH seems not to exert direct cytotoxic activity, suggesting an integrated overall anticancer action elicited by the three pyranocoumarin species together (Lü et al., 2022).

In light of this, as reported in paragraph 7, MedChem efforts were also directed to prolong decursin/DA half‐life. For instance, the replacement of the ester functional group with a thiocarbamic one gave birth to derivative DPTC (**53**, Figure 3) provided of an improved in vivo stability (Zhang, Shaik, et al., 2012).

Hopefully, future investigations will indicate the achievable concentration range and the desirable rate of the three compounds to obtain an efficient anticancer activity without affecting noncancer cells and/or semisynthetic derivatives properly modified to reach the scopes.

## 10 TOXICITY, SIDE EFFECTS, AND SAFETY OF AGN PYROCOUMARINS

AGN extract has been safely used by Asian population for long time. However, scientific studies exploring safety of AGN pyranocoumarins are very few. Yun et al. (2015) investigated the subchronic toxicity and genotoxicity in male and female rats of different doses of AGN extracts (125, 250, 500, 1000, and 2000 mg/kg body weight) after 13 weeks by gavage administration. The results evidenced no significant alterations of parameters such as food consumption, body weight, mortality, hematology, biochemistry, necropsy, organ weight, and histopathology. Thus, no significant adverse effect was evident in rats after oral intake even at the highest dose tested (2000 mg/kg/day). Additionally, genotoxic assays revealed that AG extract was not clastogenic and mutagenic. In accordance with previous studies (Kim, Lee, et al., 2009), AGN extracts also looks to be safe for oral administration.

However, the issue related to the interaction of AGN pyrocoumarins with cytochrome P450 family could be a potential concern. As reported in the previous section, decursin, DA, and DOH are reported to inhibit not only CYP2A6 and CYP2J2 activity, but also CYP1A2, thus impeding the first‐pass metabolism of the drugs substrate of the corresponding CYP isoforms. To get light on this aspect, the effects of decursin on the PK of CYP1A2 substrate theophylline were explored in rats. The LC–MS/MS analysis of theophylline and its major metabolites in blood showed PK parameters variation compatible with a reduced metabolism (decreased elimination constant, increased AUC and *C*_max_, prolonged half‐life; Chae et al., 2012). Therefore, particular attention should be paid in the concomitant consumption of AGN extract and other CYP‐metabolized drugs.

## 11 CURRENT MEDICAL APPLICATIONS

As already mentioned, AGN is one of the most popular herbal medicines in Asian countries including Korea (Lee, Lee, Jung, et al., 2003). Its roots have been used to treat female afflictions and anemia since in oriental medicine the plant is believed to be capable of tonification and activation of blood and regulation of menstruation (Lee, Lee, Jung, et al., 2003). Thus, AGN has been named “female ginseng” and has been widely used to treat blood deficiency and various diseases in women since ancient times in Korea (Sun et al., 2015). Moreover, the roots were used also as a sedative, anodyne, and tonic agent (Kumar et al., 2013; Lee, Lee, Jung, et al., 2003) for headaches, wounds, arrhythmia (Jung & Huneck, 1991), relief of pain, moistening of the intestines, and relaxation of the bowels.

In traditional medicine, AGN is usually prepared by boiling the dried root in water to extract the effective ingredients (Lü et al., 2015). At the commercial level, AGN extract or AGN‐containing herbal mixtures are sold in USA and globally as dietary supplements for pain killing, memory enhancement, and postmenopausal symptom (Lü et al., 2015).

Despite the extensive use of AGN in oriental traditional medicine and in commercial preparation, official studies in human subjects are lacking. To date, only two clinical trials are reported under the ClinicalTrials.gov identifier (NCT number) NCT02114957 and NCT05375539 at Early Phase 1 or formerly listed as Phase 0 and Phase 1, respectively. In the clinical trial NCT02114957, the researchers conducted a single oral dose human PK study of decursin, and DA delivered through an AGN‐based dietary supplement Cogni.Q (purchased from Quality‐of‐Life Labs, Purchase, NY). The study involved 20 healthy subjects, that is, 10 men and 10 women, each consuming 119 mg DOH and 77 mg DA from 4 vegicaps (Zhang et al., 2015). Plasma analysis revealed that men absorbed decursin and DA faster than women and took short time to reach the maximum concentration of DOH, supporting the conversion of decursin and DA to DOH. Notably, DA conversion occurs slightly slower in humans than in rodents (Shehzad et al., 2018; Zhang et al., 2015). Very recently, a second clinical trial was approved (NCT05375539). The Phase I study, entitled “*Angelica* herbal supplement *Angelica gigas* Nakai ‐CognI.Q acute dose safety and pharmacokinetics dose‐response in prostate cancer patients (PK Dose Trial)” aims to obtain acute dose safety and PK/PD data in a dose–response trial in prostate cancer patients. Currently, the study is recruiting participants, with an estimated enrolment of 12 participants. Notably, case documentation of the administration of a cocktail of nine herbs including AGN, in two patients affected by non‐Hodgkin lymphoma with multiple comorbidities, reported a prostate cancer antigen (PSA) suppression with a general improvement of the quality of life (Lee et al., 2021). The results of this clinical trial are extremely cheering; nevertheless, large‐scale clinical trials and in‐depth PD and PK studies are still required.

## 12 CONCLUSIONS

Nature has been effectively utilized as the primary source of drug prototypes. Over the years, many phytochemical compounds extracted from herbs have been isolated, their polypharmacology has been investigated, and many of them have entered the clinic as the therapy of choice. However, in most cases, NPs represented valid hit/lead compounds that need chemical manipulation and optimization in order to develop new synthetic or semisynthetic derivatives as potential drug candidates.

In this view, the pyrocoumarins decursin, DA, and DOH represent a very interesting pharmaceutical tools, as confirmed by the use of *A. gigas* roots in the traditional medicine of various Asian countries. However, as usual for NPs, a plethora of targets have been identified and described in the literature, together with the application in different pathological frameworks. In addition, the available information is often fragmented, suggesting the need for more fresh and complete data useful in establishing SAR. Based on established SAR, structural modification for further improvement in PD/PK profile is also possible.

From the analyzed data and our critical examination, the following conclusions can occur:

- AGN produces different coumarin derivatives variously substituted with *O*‐containing saturated/unsaturated linear/cyclic moieties.
- Many studies published in the last decades suggest the AGN natural derivatives, decursin, DA, and DOH as active against different cellular targets, often crucial in tumoral settings.
- However, the rapid and extensive CYP‐mediated metabolism of decursin and DA to DOH makes rise questions about the real in vivo involvement of identified molecular targets and should guide readers to take a critical retrospection of the cell culture studies. In light of this, new studies are required to clarify the real contribution of each compound to the global effect. Since DOH has been identified as the only active compound in vivo, comparative studies of DOH and its prodrugs and specific DOH target involvement could be of help to establish DOH pharmacodynamic; while in vivo investigations in mouse and human blood will enlighten PK aspects, including DOH metabolites and intermediates, and their implication with CYP450 (Lü et al., 2022).
- Guided by the limited activity of DOH, structural modifications were mainly oriented to the *O*‐functionalization on DOH free hydroxyl group as a tool for potency improvement against different targets. Future structure simplification investigations may evidence which portions are essential for the interaction with specific molecular targets, and more metabolically stable functional groups will be chosen to link the coumarin scaffold to the proper side chain.

Although the pyrocoumarins from AGN roots are endowed with documented anticancer effects, further comprehensive in vitro/in vivo studies and clinical trials are critical to fill the gaps in the knowledge of decursin, DA, and DOH pharmacological profiles, to allow a more efficacious clinical exploitation.

## AUTHOR CONTRIBUTIONS

**Simona Sestito:** Data curation (equal); investigation (equal); methodology (equal); supervision (equal); validation (equal); visualization (equal); writing – original draft (equal); writing – review and editing (equal). **Roberta Ibba:** Data curation (equal); investigation (equal); methodology (equal); writing – original draft (equal); writing – review and editing (equal). **Federico Riu:** Data curation (equal); investigation (equal); methodology (equal); writing – original draft (equal); writing – review and editing (equal). **Sara Carpi:** Data curation (equal); investigation (equal); methodology (equal); writing – original draft (equal); writing – review and editing (equal). **Antonio Carta:** Data curation (equal); investigation (equal); methodology (equal); writing – original draft (equal); writing – review and editing (equal). **Clementina Manera:** Data curation (equal); investigation (equal); methodology (equal); writing – original draft (equal); writing – review and editing (equal). **Solomon Habtemariam:** Data curation (equal); investigation (equal); methodology (equal); writing – original draft (equal); writing – review and editing (equal). **Balakyz Yeskaliyeva:** Data curation (equal); investigation (equal); methodology (equal); supervision (equal); validation (equal); visualization (equal); writing – original draft (equal); writing – review and editing (equal). **Zainab M. Almarhoon:** Data curation (equal); investigation (equal); methodology (equal); writing – original draft (equal); writing – review and editing (equal). **Javad Sharifi‐Rad:** Conceptualization (equal); data curation (equal); investigation (equal); methodology (equal); project administration (equal); supervision (equal); validation (equal); visualization (equal); writing – original draft (equal); writing – review and editing (equal). **Simona Rapposelli:** Data curation (equal); investigation (equal); methodology (equal); project administration (equal); supervision (equal); validation (equal); visualization (equal); writing – original draft (equal); writing – review and editing (equal).

## ACKNOWLEDGEMENTS

None.

## FUNDING INFORMATION

None.

## CONFLICT OF INTEREST STATEMENT

The authors declare that they have no competing interests.

## Data Availability

The data that support the findings of this study are available from the corresponding author upon reasonable request.
